# A practice changing paper: biallelic inactivation of BRCA2 in Fanconi anaemia

**DOI:** 10.1038/s44276-024-00054-w

**Published:** 2024-04-19

**Authors:** Diana Prepelita, Marc Tischkowitz

**Affiliations:** 1 Department of Medical Genetics, University of Medicine and Pharmacy “Carol Davila”, Bucharest, Romania; 2 Department of Medical Genetics, National Institute for Health Research Cambridge Biomedical Research Centre, University of Cambridge, Cambridge, UK

Some scientific discoveries fundamentally reshape our understanding of disease, opening up novel avenues for exploration. In 2002, Howlett et al. ushered in a new era by establishing, for the first time, the association between the *BRCA2* gene and Fanconi Anaemia [1]. The identification of biallelic pathogenic variants in *BRCA2* gene as the molecular cause of Fanconi Anaemia complementation group D1 represented a landmark discovery and 21 years later we can see how it paved the way in understanding the intricate relationship between genetic variation, complex cellular processes and disease pathogenesis.

Breast cancer (BC) is the most prevalent malignancy among women, with a subset harbouring inherently increased susceptibility due to germline pathogenic variants in the *BRCA1* and *BRCA2* genes. This understanding profoundly changed the management of BC, enabling identification of at-risk individuals, implementation of preventative measures and the development of targeted treatment.

Fanconi Anaemia (FA) is a heterogenous disease characterized by congenital and developmental abnormalities, bone marrow failure and greatly increased risks for blood and solid cancers (mainly squamous cell carcinoma), with a hallmark feature of cross-linker hypersensitivity [2]. *FANCC* was the first FA gene to be identified in 1992, subsequently, other genes in the same molecular pathway were identified, leading to the realisation that FA was both clinically and genetically heterogeneous [3].

In 2002, there were at least eight distinct complementation groups of FA (A, B, C, D1, D2, E, F, G) defined by somatic cell fusion studies and six FA genes that have been cloned (A, C, D2, E, F, G). The underlying genetic cause for FA-B and FA-D1 was unknown [1]. In 2001, Garcia-Higuera et al. published a paper suggesting that BRCA genes and FANC genes appeared to be part of the same molecular pathway [4]. At that time, several studies performed on mice showed that biallelic knock-out of *BRCA2* (BRCA2^-/-^) led to embryonic lethality [5]. Given the importance of BRCA genes in DNA damage repair, these observations were consistent with the prevailing dogma at the time that biallelic inactivating variants in *BRCA1* or *BRCA2* would be incompatible with life.

The landmark paper, published by Howlett et al. in 2002, was the first to identify bilallelic *BRCA2* pathogenic variants in FA-D1 cell lines. The HSC62 cell line had a biallelic homozygous IVS19-1G > A pathogenic variant that was predicted to lead to loss of exon 20 and the EUFA423 cell line was compound heterozygous for 7691insAT and 9900insA *BRCA2* pathogenic variants in exon 27, both frameshift and predicted to disrupt the protein structure [1].

This laboratory-based study was rapidly validated by clinical studies. In 2003, Offit et al. investigated four kindreds with FA, some of them also presenting with extensive family history of BC, and identified biallelic pathogenic variants in *BRCA2* gene [6]. A year later, Wagner et al. reported seven cases with FA-D1, extending the phenotype and correlating the presence of biallelic *BRCA2* pathogenic variants with a worse prognosis type of FA, due to the higher prevalence of acute myeloid leukaemia at a younger age (at age 5, 5.41% in BRCA2-FA vs. 1% in non-BRCA2-FA) and occurrence of solid tumours, such as Wilms tumour [7]. It thus become apparent that while monoallelic pathogenic variants in *BRCA2* gene led to an increased risk of breast, ovarian, prostate and pancreatic cancer, other tissues are at increased cancer risk when biallelic pathogenic variants are present.

Uncovering the connection between the biallelic pathogenic variants in *BRCA2* gene and FA was an unexpected discovery which prompted researchers to further explore potential cancer associations in the area of DNA repair by homologous recombination. The striking observation that a gene can elicit markedly distinct phenotypes when inherited in a different fashion changed the thinking on FA and suddenly a rare paediatric congenital anomaly syndrome became front and centre in the breast cancer research field.

Using the same principles, other genes were discovered as dual contributors to both dominant breast and/or ovarian cancer susceptibility and recessive FA. First, monoallelic *BRIP1* pathogenic gene variants were found to be associated with an increased risk of breast cancer, while biallelic variants in *BRIP1* were proved to be responsible for FA complementation group J [8, 9]. Later the association with breast cancer turned out to be incorrect [10], but *BRIP1* is an important ovarian cancer predisposition gene. Following the initial discovery of *PALB2* in 2006 [11], two groups independently demonstrated the presence of *PALB2* pathogenic variants in affected individuals and defined a new subtype of FA, designated FA-N [11–13]. *PALB2* was subsequently confirmed to be a major breast cancer susceptibility gene [14].

Due to the intimate interconnection of the FANC and BRCC molecular complex, certain genes had long been suspected to operate in a similar fashion. However, the rarity of FA subtypes within the population required further studies to generate evidence to conclusively establish these connections. In 2010, Meindl et al. demonstrated for the first time that monoallelic *RAD51C* variants confer an increased risk for BC and at a similar time-point Vaz et al. associated biallelic variants with FA subtype FA-O. Interestingly, *RAD51C* gene does not seem to associate with haematological disease [15, 16]. In 2012, Domchek et al. identified biallelic hypomorphic *BRCA1* alleles in a 28 year-old woman with an FA-like phenotype [17]. Another similar report appeared from Sawyer et al. in 2014, confirming that the *BRCA1* gene was FA-S [18]. Exploration of genes that are part of the FA pathway is an ongoing process and led to the identification of 22 disease-causing genes to date [19].

Understanding the molecular machinery of this complex pathway led to the hypothesis that homologous repair deficient (HRD) cancers can be sensitive to platinum and furthered the development of a new class of targeted therapy that introduced the concept of synthetic lethality. It was presumed that HRD cancers can be pushed to an extreme level of genetic instability, that would be highly detrimental to the cell’s fate. Molecules that inhibit poly ADP-ribose polymerase (PARPi), a family of proteins involved in stress response, chromatin remodelling, DNA repair and apoptosis, were first approved for the treatment of BRCA-associated ovarian cancer in 2014 and proved to be a success. This class of molecules continues to be developed to this day [20].

To conclude, the paper published by Howlett et al. in 2002 was an important breakthrough that connected a well-known breast cancer predisposition gene to a rare paediatric syndrome at the molecular level, which in turn opened up new therapeutic targets. It is an example of how a discovery can catalyse a paradigm shift and serve as foundation for future scientific endeavours.

## Data Availability

No datasets were generated or analysed during the current study.
